# Protein Microarray Analysis of Antibody Responses to *Plasmodium falciparum* in Western Kenyan Highland Sites with Differing Transmission Levels

**DOI:** 10.1371/journal.pone.0082246

**Published:** 2013-12-02

**Authors:** Elisabeth Baum, Kingsley Badu, Douglas M. Molina, Xiaowu Liang, Philip L. Felgner, Guiyun Yan

**Affiliations:** 1 Department of Medicine, Division of Infectious Diseases, University of California Irvine, Irvine, California, United States of America; 2 Department of Immunology, Noguchi Memorial Institute for Medical Sciences, College of Health Science, University of Ghana, Accra, Ghana; 3 Antigen Discovery Inc., Irvine, California, United States of America; 4 Program in Public Health, University of California Irvine, Irvine, California, United States of America; Bernhard Nocht Institute for Tropical Medicine, Germany

## Abstract

Malaria represents a major public health problem in Africa. In the East African highlands, the high-altitude areas were previously considered too cold to support vector population and parasite transmission, rendering the region particularly prone to epidemic malaria due to the lack of protective immunity of the population. Since the 1980’s, frequent malaria epidemics have been reported and these successive outbreaks may have generated some immunity against *Plasmodium falciparum* amongst the highland residents. Serological studies reveal indirect evidence of human exposure to the parasite, and can reliably assess prevalence of exposure and transmission intensity in an endemic area. However, the vast majority of serological studies of malaria have been, hereto, limited to a small number of the parasite’s antigens. We surveyed and compared the antibody response profiles of age-stratified sera from residents of two endemic areas in the western Kenyan highlands with differing malaria transmission intensities, during two distinct seasons, against 854 polypeptides of *P. falciparum* using high-throughput proteomic microarray technology. We identified 107 proteins as serum antibody targets, which were then characterized for their gene ontology biological process and cellular component of the parasite, and showed significant enrichment for categories related to immune evasion, pathogenesis and expression on the host’s cell and parasite’s surface. Additionally, we calculated age-fitted annual seroconversion rates for the immunogenic proteins, and contrasted the age-dependent antibody acquisition for those antigens between the two sampling sites. We observed highly immunogenic antigens that produce stable antibody responses from early age in both sites, as well as less immunogenic proteins that require repeated exposure for stable responses to develop and produce different seroconversion rates between sites. We propose that a combination of highly and less immunogenic proteins could be used in serological surveys to detect differences in malaria transmission levels, distinguishing sites of unstable and stable transmission.

## Background

Malaria represents a major public health problem in Africa [1]. In the East African highlands, even in high-altitude areas previously considered too cold to support vector population and parasite transmission [2], frequent malaria epidemics have been reported since the 1980’s [3]. *Plasmodium falciparum* infections have been detected in areas as high as 1,600-2,400m above sea level in Africa [4], where there is a marked gradient of parasite prevalence along the altitude transect [5-7]. Prior to the 20^th^ century, there was no or negligible malaria in the African highlands [8,9] and successful intensive malaria control efforts put in place after the recent outbreaks have reduced malaria prevalence and incidence in the region [10], rendering the East African highlands particularly prone to epidemic malaria due to the lack of the protective immunity, and causing significant human mortality amongst all age groups [11]. Therefore, malaria transmission monitoring in the East African highlands is an important public health problem.

Despite the overall lower immunity of the population in these historically malaria-free areas, the many successive outbreaks since the 1980’s may have generated some level of immunity against *P. falciparum* amongst highland residents. The antibody response to *Plasmodium* is cumulative and long lasting, developing after repeated exposures to the parasite and persisting for months or years after infection has resolved [12]. The antibody response to *Plasmodium* varies amongst individuals of different age groups (i.e. toddlers, older children and adults) as well as amongst individuals of same age groups from areas of different parasite prevalence [13]. The repertoire of targets of the antibody response also expands after multiple infections, with the number of recognized antigens being correlated to parasite prevalence, the host’s age and immunity to clinical malaria [14-16]. Serological studies bring forth indirect evidence of human exposure to the parasite, and can reliably assess prevalence of exposure and transmission intensity in an endemic area [17-19]. However, the vast majority of serological surveys for malaria infection have been, hereto, limited to a small number of the parasite’s antigens. 

The work we present here is an expansion of the study published by Badu et al. [20], in which the antibody response to the 19kDa fragment of merozoite surface protein-1 (MSP-1_19_) of *P. falciparum* was examined in populations from two endemic areas in the western Kenyan highlands. There, the tremendous variations of malaria transmission intensity in a small spatial scale are caused by substantial differences in altitude, topography and other environmental conditions [6,7,21,22]. We now expand our antibody profiling survey to include 854 *P. falciparum* polypeptides by using protein microarray technology. This platform provides an efficient time- and cost-effective method for high-throughput screening of open-reading frames from genomic databases for antigen identification for vaccine or diagnostics, and the unique methodology for construction and the many attributes of this platform were reviewed by Doolan [23]. Protein microarrays have been used to explore the humoral response to several human pathogens [24-28], including *P. falciparum* [15,29-33]. 

The objectives of our study were two-fold: firstly, to identify the targets of broad antibody responses to *P. falciparum* and compare the reactivity profiles amongst residents of sites with differing parasite prevalence; and secondly, identify new serological markers sensitive to differing malaria transmission intensities to be used in serological surveys to determine the relative parasite transmission levels amongst sites in areas of heterogeneous risk of malaria. To this end, we surveyed the antibody response profiles of age-stratified sera from residents of two endemic areas in the western Kenyan highlands differing in malaria transmission intensities, during two distinct seasons, against 854 *P. falciparum* polypeptides, and analyzed the effects of age, season and parasite transmission level on intensity and breadth of antibody responses. We then calculated seroconversion rates for the immunogenic polypeptides and identified antigenic markers sensitive to variations in parasite transmission levels amongst sites, which could be used as serological markers for estimation of the relative levels of parasite burden in endemic areas, distinguishing sites of unstable and stable transmission. 

## Materials and Methods

### Study sites

The study sites were previously described by Badu et al. [20] and Githeko et al. [34]. Briefly, four villages of Kakamega county, in the western Kenyan highlands, were selected for the study. The topography of the highlands comprises hills, valleys and plateaus. Rivers and streams run along the valley bottoms and swamps are a common feature, providing stable vector-breeding habitat, where about 90% of adult mosquito population aggregates within a distance of 300m from the valley bottoms [35]. In contrast, the hillside gradients produce efficient drainage and hilltop sites provide less stable vector-breeding habitats. Two villages, Iguhu and Lidambiza were defined as valley bottom sites (altitude, ~1,400 m) while Sigalagala and Museno villages were defined as hilltop (~1,600 m) locations. The distance between the valley bottom and the hilltop sites, by road, is approximately 4.5 km. *Plasmodium falciparum* prevalence is 2-fold higher in the valley bottom than in the hilltop sites [20], and infection prevalence amongst school children was shown to be 68% in the valley bottom and 26.7% in the hilltop [34]. In Kenya the rainfall is seasonally bimodal with the first and longer rainy season beginning April-May and ending in July. The second and shorter rainy season occurs in September through October. Thus November through February are known to be dry with little or no rains and cool temperature [36].

### Sera

A subset of leftover serum samples from a cross-sectional study published by Badu et al. [20] were selected based on donor age, season of collection and site of residence of donors to form 12 sera groups, comprising a maximum number of 10 randomly selected samples per group. Where the number of available samples for a sera group was smaller than 10, all samples at-hand were included. Serum was collected from consenting participants residing either on the valley bottom or hilltop locations, during the main dry and wet seasons of 2009. Dry season samples were collected from the 3rd of March to the 3rd of April; rainy season samples were collected about 6 weeks from the onset of rains, between 16th of June to 23rd of August.  Malaria cases peak 2-3 months after peaks of rainfall [37]; so typically a peak in rainfall in April-May is followed by a distinct peak in malaria incidence in June. Serum samples from 108 individuals were divided into 12 study sera groups according to age (age brackets: 0 to 4, 5 to 14 and >15 years old), residence site (valley bottom or hilltop) and season (wet or dry). In general, 10 serum samples were tested for each group, with the exception of groups formed by serum collected at the hilltop site during dry season from 0-4 (3 sera tested), 5-14 (7 sera) year olds, and during wet season from 5-14 years old (8 sera); sera from 10 adult blood donors from the U.S. without history of travel to malaria endemic regions were used as unexposed controls.

### Sample size justification and statistical power

To determine the seroprevalence rate of the residents in uphill and valley to the antigens in the Protein array, we used a total of 108 samples (48 in hilltop and 60 in valley). This sample size enabled us to detect 15% margin of error in seroprevalence rate within each group, using alpha 0.05. This sample size also enabled us to determine a difference of seroprevalence rate of 0.25 with a power of 0.8, using one-sided test and alpha of 0.05.

### Ethics statement

Sera from human donors were originally collected for other studies [20] for which informed consent had been obtained; patient identifier information had been removed. The study was approved by the Kenya Medical Research Institute Ethical Review Committee [SCC No. 1382(N)] and the Institutional Review Board of the University of California, Irvine.

### *P. falciparum* protein microarray

A protein microarray of *P. falciparum* (*Pf*) reactive antigens (Antigen Discovery Inc., Irvine, CA) displaying 854 sequence-verified polypeptides printed as *in vitro* transcription translation (IVTT) reactions as described in Davies et al. [38] was used. Quality control of array slides revealed over 98% protein expression efficiency of *in vitro* reactions spotted. Protein amount was consistent between multiple subarrays, with signal intensity of anti-6xHis tag probing showing R^2^=0.93 between subarrays and slides. The protein targets on this array were down-selected from larger microarray studies [15,29,33], based on immunogenicity and antigenicity to humans. Due to gene length, some proteins were printed on the microarray in multiple spots of overlapping polypeptides [33]. Protein array platform information is deposited in NCBI's Gene Expression Omnibus [39] (http://www.ncbi.nlm.nih.gov/geo/) and is accessible through GEO Platform accession number GPL17312. Figure S1 shows the distribution of parasite life stage of maximum expression for the proteins spotted on the microarray, based on information from PlasmoDB. For probing, serum samples were diluted 1:200 in Protein Array Blocking buffer (Whatman Inc, Sanford, ME) supplemented with 10% (vol/vol) DH5α *E. coli* lysate (MCLAB, San Francisco, CA) and incubated on arrays over-night at 4°C. Microarray slides were incubated in biotin SP-conjugated affini-pure goat anti-human IgG Fc-fragment specific secondary antibody (Jackson ImmunoResearch, West Grove, PA) diluted 1:200 in blocking buffer, and detected by incubation with streptavidin-conjugated SureLight P-3 (Columbia Biosciences, Columbia, MD). The slides were washed and air-dried by brief centrifugation. Probed array slides were scanned in a Perkin Elmer ScanArray Express HT at a wavelength of 670 nm, at 95% laser power and 55% PMT. The output grey scale TIFF files generated by the scanner were quantitated using ProScanArray Express software (Perkin Elmer, Waltham, MA) with spot-specific background correction. 

### Array data

The array data is publicly available through NCBI's Gene Expression Omnibus and is accessible through GEO Series accession number GSE48089.

### *P. falciparum* gene annotation

Annotation of genes presented in this study follow gene accession numbers published on PlasmoDB (http://plasmodb.org/plasmo/) [40]. ORF ID accession numbers corresponding between PlasmoDB and GenBank databases are presented in the array data file deposited in the GEO Series above. 

### Data analysis

For analysis of antibody binding to *Pf* polypeptides on the microarray the following steps were taken: (i) the mean background signal of antibody binding to 31 control spots of IVTT reaction without DNA template (*no DNA* spots) were subtracted from each sera’s raw values of antibody binding measured as the mean signal intensity of spots of printed polypeptides; negative or zero values after background subtraction were assigned a net value of 0. Data normalization was achieved by log_10_-transformation of net values. Normalized data was used for statistical analyses; for figure representations of the data the anti-log_10_ of the net values was used; (ii) the average and 95% confidence intervals (CI) of antibody binding signal intensity for each study and control groups were calculated for each polypeptide on the array. For proteins printed as multiple overlapping polypeptides, the averaged signal for all fragments was used in analysis of breadth and intensity of response; for z-score and seroconversion rate calculations, each polypeptide was considered individually. 

Protein targets were considered immunogenic if the following criteria were satisfied for at least one study sera group: (i) for each sera group, the average signal intensity to the protein had to be above the group’s upper CI value of signal intensity of *no DNA* spots; and (ii) the study group’s lower confidence interval for signal intensity did not overlap with the control group’s upper confidence interval for the protein spot. Individual serum samples were considered seropositive for a protein or polypeptide if the sample’s signal intensity value was above the upper CI value of signal intensity for the unexposed control group. 

The following analyses were performed on data of antibody binding to proteins considered immunogenic only: for analysis of intensity of response, (i) ANOVA testing with *post hoc* Tukey-Kramer (Tukey’s Honestly Significant Difference, HSD) and Student’s T-test analysis were used for pairwise comparison of the mean signal intensity amongst the study groups using JMP9.0; significance tests were 2-sided and set at the 0.05 level for type I error; and (ii) z-scores were calculated as the number of standard deviations above or below the mean of the control group. For comparison of breadth of response amongst sera groups, (iii) two-tailed large-sample proportion hypothesis testing as in McClave and Sincich, 2012 [41] was used. (iv) Annual rates of seroconversion and reversion to seronegativity for the immunogenic polypeptides were calculated using Systat 11 (Systat Software, Chicago, IL) by fitting age-specific seroprevalence data to a reversible catalytic model using the maximum-likelihood method that assumes binomial error distribution: *P*_*t*_=λ/λ+ρ(1-*e*(-(λ+ρ)*t)) [17]; where *P*_*t*_ is the proportion of seropositive individuals in each age group *t*, λ is the annual rate of seroconversion and ρ is the annual rate of reversion to seronegativity. Models were fitted independently for the hilltop and valley bottom locations. Seroprevalence was calculated using 5 (hilltop) and 6 (valley bottom) age groups, and excluded individuals below 2 years of age. (v) Mann-Whitney U test was used to compare seroconversion rate values between sites.

Enrichment analysis for gene ontology categories for Biological Process and Cellular Component was performed using annotated GO Process and GO Component entries from PlasmoDB.org database. 

## Results

### Characterization of *P. falciparum* antigenic targets

Of 854 targets on the array, 119 polypeptides comprising 107 unique *P. falciparum* (*Pf*) proteins were considered immunogenic to at least one of 12 study groups when compared to unexposed controls. The PlasmoDB gene ID and function of the immunogenic proteins are listed on Table S1. Seventy-one of the 107 proteins had been previously reported as immunogenic by other protein microarray studies of naturally exposed [15,31,33] and protected attenuated-sporozoite vaccinees [29] and are identified in Table S1. Amongst the most frequently recognized antigens are several members of early transcribed membrane protein (ETRAMP) family, the merozoite surface protein (MSP) family, several PfEMP1 proteins and liver stage antigen (LSA1 and 3) proteins. 

Gene ontology annotation for Cellular Component, Biological Process and life cycle stage of maximum expression were obtained from PlasmoDB for all proteins displayed on the microarray including the immunogenic proteins recognized by study sera. Charts showing the distribution of life cycle stage of maximum expression, the biological components and cellular processes in which the immunogenic proteins are involved in are shown in Figure S2. Enrichment analysis of gene ontology annotations showed that, compared to all targets available on the array, there was significant enrichment to certain categories amongst the immunogenic proteins, as shown in Table S2. The enriched Cellular Component categories in which the immunogenic proteins were expressed were ‘host cell-related’, ‘parasitophorous vacuole’, the parasite’s ‘cell surface’ and ‘membrane’, and ‘Maurer’s cleft’. The enriched Biological Process categories were ‘antigenic variation’, ‘entry into host cell’, ‘cell-cell adhesion’, and ‘attachment of GPI anchor to protein’. Table 1 presents the immunogenic proteins recognized by study sera corresponding to each of the enriched gene ontology categories. The 8 *var* gene products (PfEMP1) recognized by sera were present in the enriched categories for both Component (‘host cell-related’) and Process (‘antigenic variation’); MSP1, MSP7, DBLMSP, MTRAP, DBL-like protein Pf332 and the conserved *Pf* protein PF3D7_1136200 were also represented in the enriched categories for both Cellular Component and Biological Process.

**Table 1:** Enriched gene ontology categories for Cellular Component and Biological Process, and representative *P. falciparum* proteins considered immunogenic by study sera.

**Cellular Component Category**	**Immunogenic Proteins in Category**
Host cell-related	PfEMP1 (8 *var* gene products); TryThrA
Parasitophorous vacuole-related	SERA4, SERA5; MDV1; EXP2
Cell surface-related	STARP; MSP11; MTRAP; DBLMSP
Maurer's cleft-related	DBL-like protein Pf332; PIESP2; MC-2TM; ETRAMP10.2
Parasite membrane	LSA1, LSA3; ETRAMP 10, ETRAMP 14; MSP1, MSP7; CRA; PF70; ATP4; RON2; PHISTb (3 genes); conserved Pf proteins PF3D7_0407800, PF3D7_1473000, PF3D7_1302000, PF3D7_1328300, PF3D7_1136200
**Biological Process Category**	**Immunogenic Proteins in Category**
Antigenic variation-related	PfEMP1 (8 *var* gene products)
Entry into host cell	MTRAP; MSP7; EBA175; RH2b
Cell-cell adhesion, pathogenesis	EBA181; DBLMSP; DBL-like protein Pf332
Attachment of GPI anchor to protein	MSP1, MSP2, MSP5, MSP10; conserved *Pf* protein PF3D7_1136200

There was no significant enrichment for life cycle stage of maximum expression. 

### Comparison of breadth of antibody response to immunogenic *P. falciparum* proteins

**Figure 1:**
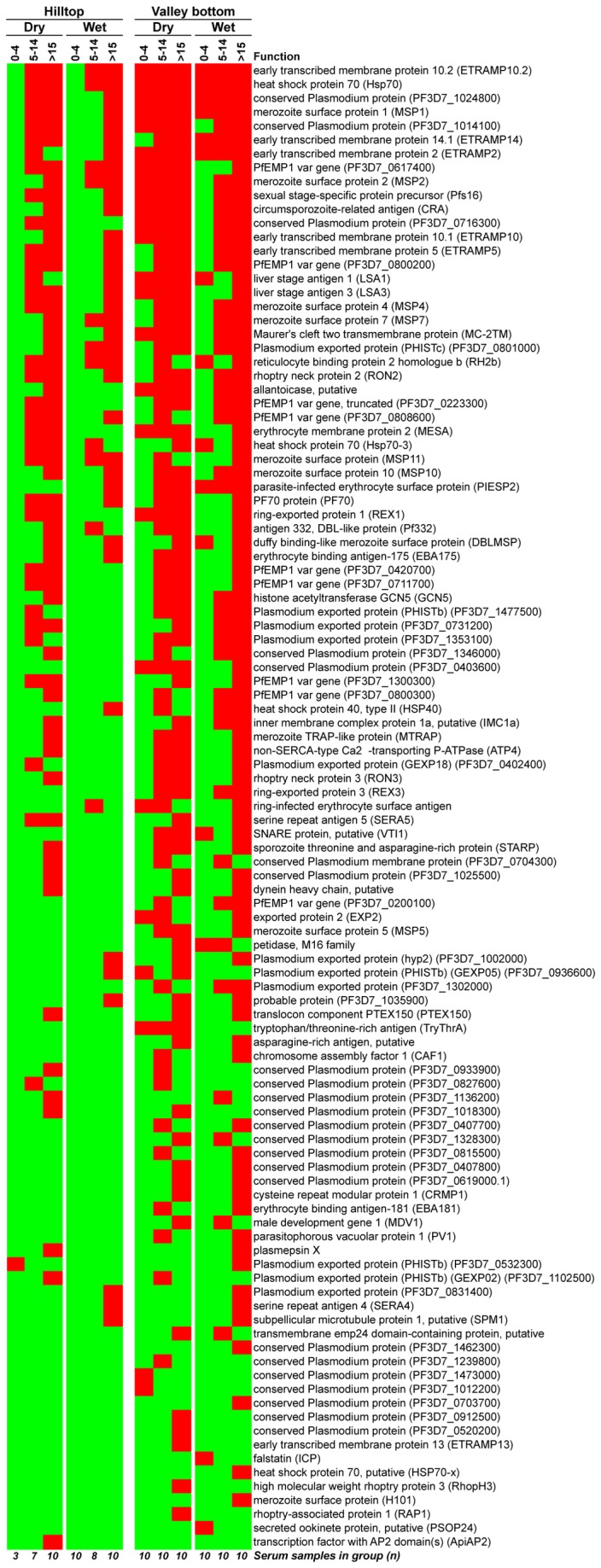
Breadth of antibody response to immunogenic *P. falciparum* proteins by sera from study groups. The binary heatmap shows positive antibody responses (red cells) and the lack of antibody binding (green cells) to 107 proteins considered immunogenic by sera from naturally exposed individuals from the Kenyan highlands. Sera cohorts are stratified by site and season of sample collection, and age of sample donors (0-4, 5-14 and >15 years old). Proteins are listed by frequency of response amongst all sera groups; description of the function and in some cases the gene ID for the ORF of each protein is shown. The number of serum samples tested in each sera group is provided on the bottom of the heatmap.

Figure 1 shows a binary heatmap of antibody reactivity to the immunogenic proteins, sorted by frequency of response, where red cells indicate antibody response to the protein by the sera group and green cells lack thereof. The most frequently recognized proteins were Hsp70, ETRAMP10.2, MSP1, and conserved *Plasmodium* protein PF3D7_1024800, which were recognized by at least 9 of the 12 sera groups. 

**Figure 2:**
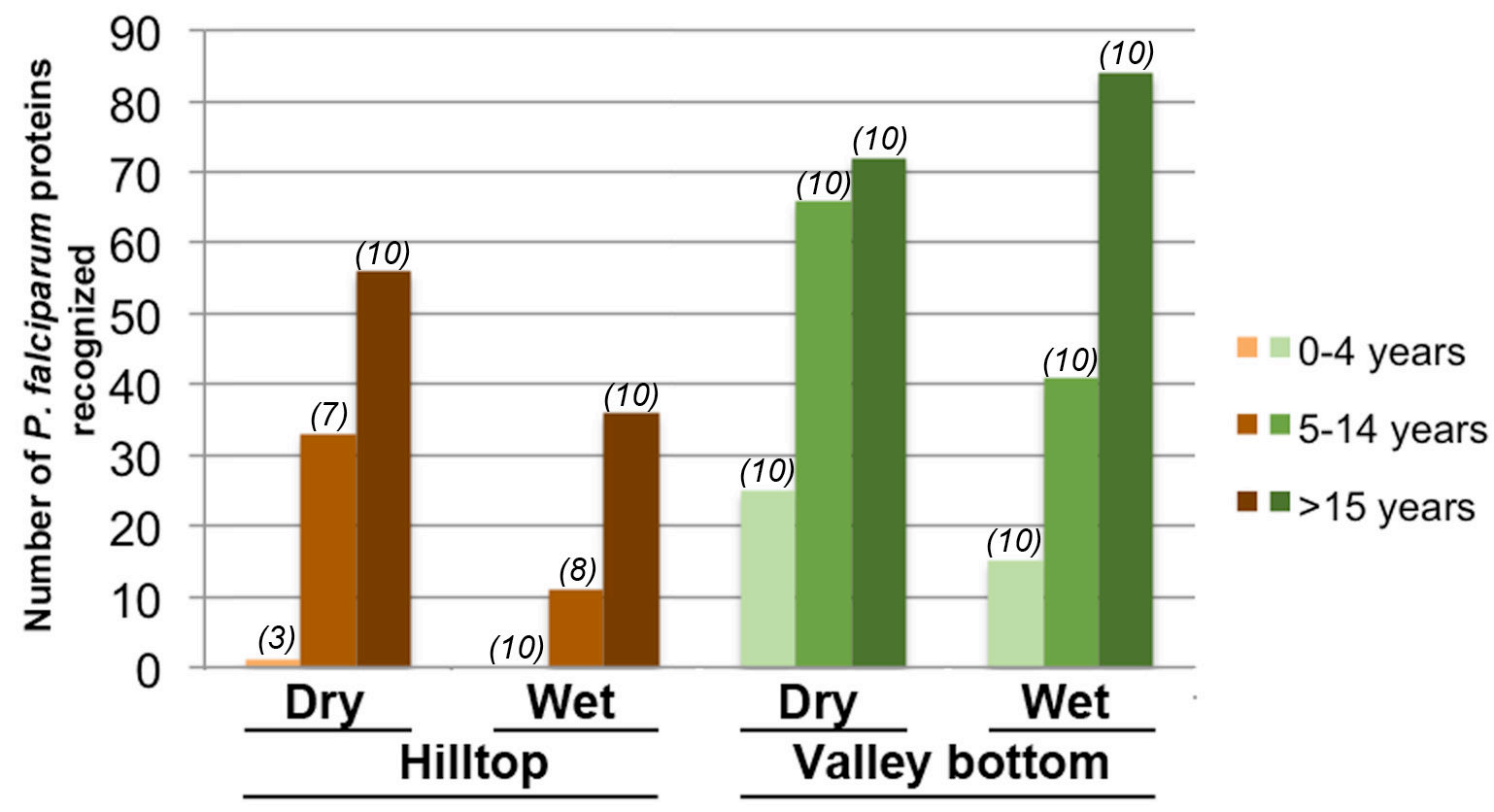
Breadth of antibody binding to immunogenic *P. falciparum* proteins by study sera groups. The number of proteins considered immunogenic by the study sera (Y axis) is plotted against sera cohorts stratified by age (0-4, 5-14 and >15 years old), season (wet and dry) and site (hilltop and valley bottom) of sample collection (X axis). The number of serum samples tested in each sera group is provided in parenthesis above the corresponding bar.

Figure 2 summarizes the number of proteins recognized by sera from each of the study groups. Sera from the hilltop reacted to a total of 73 proteins, while sera from the valley bottom reacted to 105 (proportion testing p=1.0). The breadth of the antibody response did not change with the season of sampling in the valley bottom (p=0.5); however, in the hilltop samples, there was significant difference between the number of proteins recognized by sera collected in the dry season (64) and following wet season (39) (p<.001). This significant drop in the breadth of response was observed in both 5-14 years old (y.o.) and >15 y.o. sera groups (p<.001 for either group). This is possibly an affect of the average age in the dry and wet season cohorts. For the hilltop site, the average age of donors for the dry season sampling was 2 years older than in the wet season for the 5-14 y.o. group, and 4 years for the adults. Hence individuals sampled in the dry season were generally more ‘malaria-experienced’ than those sampled in the wet season. Such difference in age was not present amongst the valley bottom samples. Additionally, abnormally heavy rains were observed in January 2009 [42], 2 months before the dry season when samples were collected for the study, causing a higher than usual parasite prevalence during that dry season, which may have contributed to stronger antibody responses to *P. falciparum*. 

When study sites are compared, there was significant difference in the breadth of antibody response only amongst the youngest age group (0-4 y.o.), with sera from the valley bottom reacting to 30 unique proteins while sera from the hilltop reacted to only 1 protein (p>.001). There was no difference in the breadth of response between sites amongst 5-14 y.o. (p=0.45) and >15 y.o. (p=0.99) sera groups. Significant differences in the number of recognized proteins were observed only between toddlers 0-4 y.o. and the other two older groups (p<0.03), for sera from both hilltop and valley bottom sites. 

### Comparison of intensity of antibody response to immunogenic *P. falciparum* proteins

**Figure 3:**
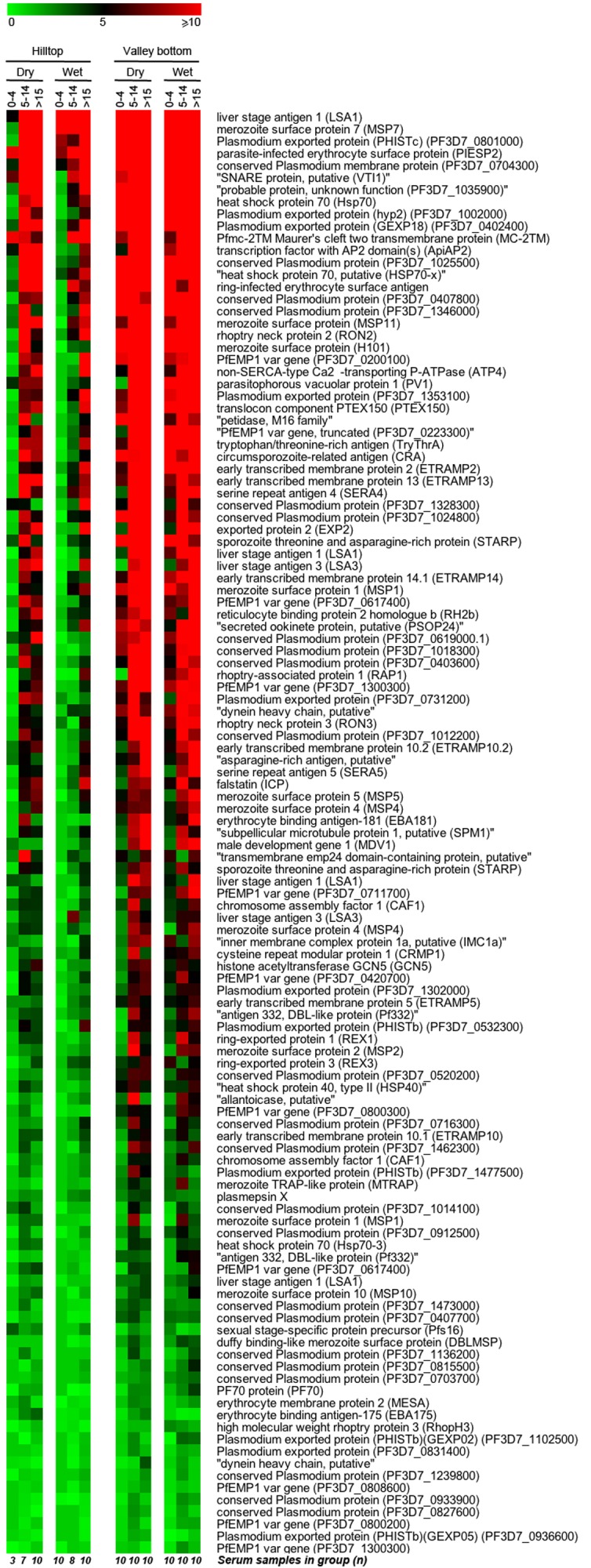
Heatmap of intensity of antibody binding to immunogenic *P. falciparum* proteins by sera from study groups. The gradient heatmap depicts z-scores of signal intensity values of antibody binding to 107 proteins considered immunogenic by sera from naturally exposed individuals from the Kenyan highlands. Gradient colors indicate the range of z-score values as the number of standard deviations above the mean of unexposed controls from 0 (green) to 5 (black) to 10 or more (red). Sera cohorts are stratified by site and season of sample collection, and age of sample donors (0-4, 5-14 and >15 years old). Proteins are listed by averaged intensity of response amongst all sera groups; description of the function and in some cases the gene ID for the ORF of each protein is shown. The number of serum samples tested in each sera group is provided on the bottom of the heatmap.

Figure 3 shows a heatmap of the z-scores of antibody binding intensity to the immunogenic proteins by the study groups, arranged by averaged z-score. The colored gradient indicates the number of standard deviations above the average signal intensity (SI) of unexposed controls. 

**Figure 4:**
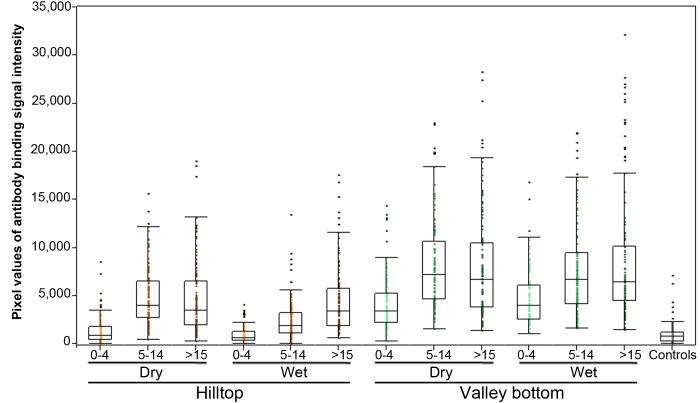
Intensity of antibody binding to immunogenic *P. falciparum* proteins by study and control sera. The mean signal intensity value of antibody binding to 107 immunogenic proteins (Y axis) is plotted against sera cohorts stratified by age (0-4, 5-14 and >15 years old), season (wet and dry) and site (hilltop and valley bottom) of sample collection (X axis) in a box-whisker plot. The lower and upper edges of the box indicate the first and the third quartiles, the line in the middle represents the median. The 1.5X interquartile range is indicated by the vertical line outside the boxes. Outlying values are indicated by dots outside boxes.

The SI of antibody binding to all 107 immunogenic proteins by the study groups are shown in the whisker-box plot in Figure 4. For the study groups, the average SI values and 95% confidence intervals (Table S3) ranged from 965.3 [817.9 to 1,112.7] for hilltop toddlers (0-4 y.o.) during wet season, to 8,818.3 [8,289.8 to 9,346.8] for valley adults (>15 y.o.), also in the wet season; the control group consisting of unexposed adult blood donors from United States had the lowest mean, 750.6 [363.3 to 1137.9]. Overall, sera from the hilltop site showed significantly lower intensity of antibody binding to the immunogenic proteins (average 3,319.5 [3,182.1 to 3,457]) than sera from the valley bottom site (6,967.7 [6,769.4 to 7,166]) (Mann-Whitney U test p<.0001). 

As with the breadth of the response, the season of sample collection affected the intensity of the antibody response in residents from the hilltop but not from the valley bottom. Sera collected at the valley bottom site, where *Pf* transmission levels are higher, showed no significant difference in intensity of antibody response between the dry (mean 6,680.2 [6,583.7 to 7,136.6]) and the following wet (7,075.3 [6,790.8 to 7,359.8]) seasons (Tukey-Kramer HSD p = 0.72; Student’s T test p = 0.30). However, sera from the hilltop residents produced significantly stronger antibody reactions to *Pf* proteins in the dry season (mean 4,339.1 [4,101.3 to 4,577]) than in the following wet season (2,638.9 [2,477.4 to 2,800.4]) (HSD and Student’s T test p = <.0001). The possible explanations for this effect have been discussed above.

**Figure 5:**
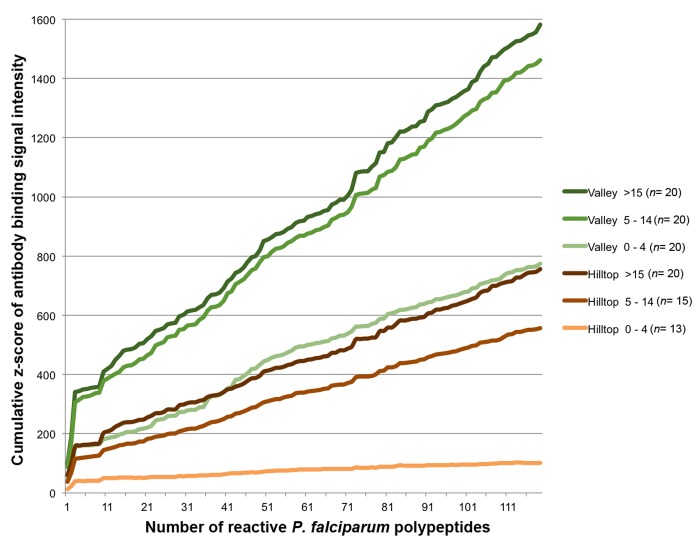
Cumulative z-scores of intensity of antibody binding to immunogenic proteins. The z-scores for signal intensity of antibody binding by age-stratified sera from the hilltop and valley bottom sites were progressively summed (Y axis) and plotted against each of the 119 immunogenic polypeptides (X axis) recognized on the microarray. Lines in shades of green represent sera from the valley bottom; lines in shades of brown, from the hilltop. The number (*n*) of samples in each sera group is shown in the plot legend.

Pronounced differences in age of acquisition of antibody response to *Pf* between residents of sites with differing parasite prevalence is clearly demonstrated in Figure 5. The z-scores for SI of antibody binding to all 119 immunogenic polypeptides were progressively summed and plotted for sera samples grouped by age and residing site. Serum samples from toddlers (0-4 y.o.) showed the most contrasting patterns: while sera from the hilltop have the lowest intensity of antibody response of all groups, sera from valley bottom toddlers show similar binding intensity as sera from adults of hilltop sites, as seen at the maxima of summed z-scores. 

Based on the z-score values, a pairwise Tukey-Kramer HSD analysis grouped the intensity of antibody response of all sera into 3 significantly distinct clusters: I) sera from toddlers 0-4 y.o. from hilltop site; II) sera from children 5-14 y.o. and adults >15 y.o. from the hilltop along with toddlers 0-4 y.o. from the valley site; and III) children 5-14 y.o. and adults >15 y.o. from the valley bottom. Table S4 shows the mean and 95% confidence intervals z-scores for each sera group, as well as a matrix of p-values for pairwise comparisons of z-scores of antibody binding intensity between sera groups. Once again, the intensity of antibody response of sera from valley toddlers was identical to that of adults from the hilltop site (p = 1.0). The next two highest correlation values were for samples of different age groups but from the same site, i.e., children and adults from the valley (p = 0.97) and from the hilltop (p = 0.78). 

### Seroconversion rates and markers of transmission variation

A simple catalytic model of age-fitted seroprevalence curves was used to calculate annual seroconversion (SCR) and reversion (SRR) rates to the immunogenic polypeptides recognized by the study sera from valley bottom and hilltop sites, and the values are presented in Table S5. Of the 119 polypeptides (representing 107 proteins) considered immunogenic by the study sera on the microarray, SCR and SRR values were obtained for 114 and 102 of those polypeptides using hilltop sera and valley bottom sera, respectively; paired values for both sites were obtained for 98 of those. The inability to calculate SCR and SRR values for all 119 immunogenic polypeptides was likely caused by the small sample size and the high seropositivity in the youngest age strata, especially in the higher transmission sites of the valley bottom, to highly immunogenic polypeptides such as MSP-1. On the microarray, MSP-1 was present as two overlapping polypeptides, one comprising residues 1-870 and the other residues 752-1721. Seropositivity to residues 1-870 was higher than to the distal residues, especially amongst valley sera where the youngest age strata showed 100% seroprevalence to MSP-1_(1-870)_ and such seroprevalence continued almost unchanged through the older age strata groups (Figure S3). Lack of change in seroprevalence rates amongst the age strata introduced confounding factors into the modeling used to calculate SCR and SRR, generating values outside the acceptable range (≤1.0); so in this study, polypeptides that generated out-of range values were reported as N/A (not available) in Table S5.

**Figure 6:**
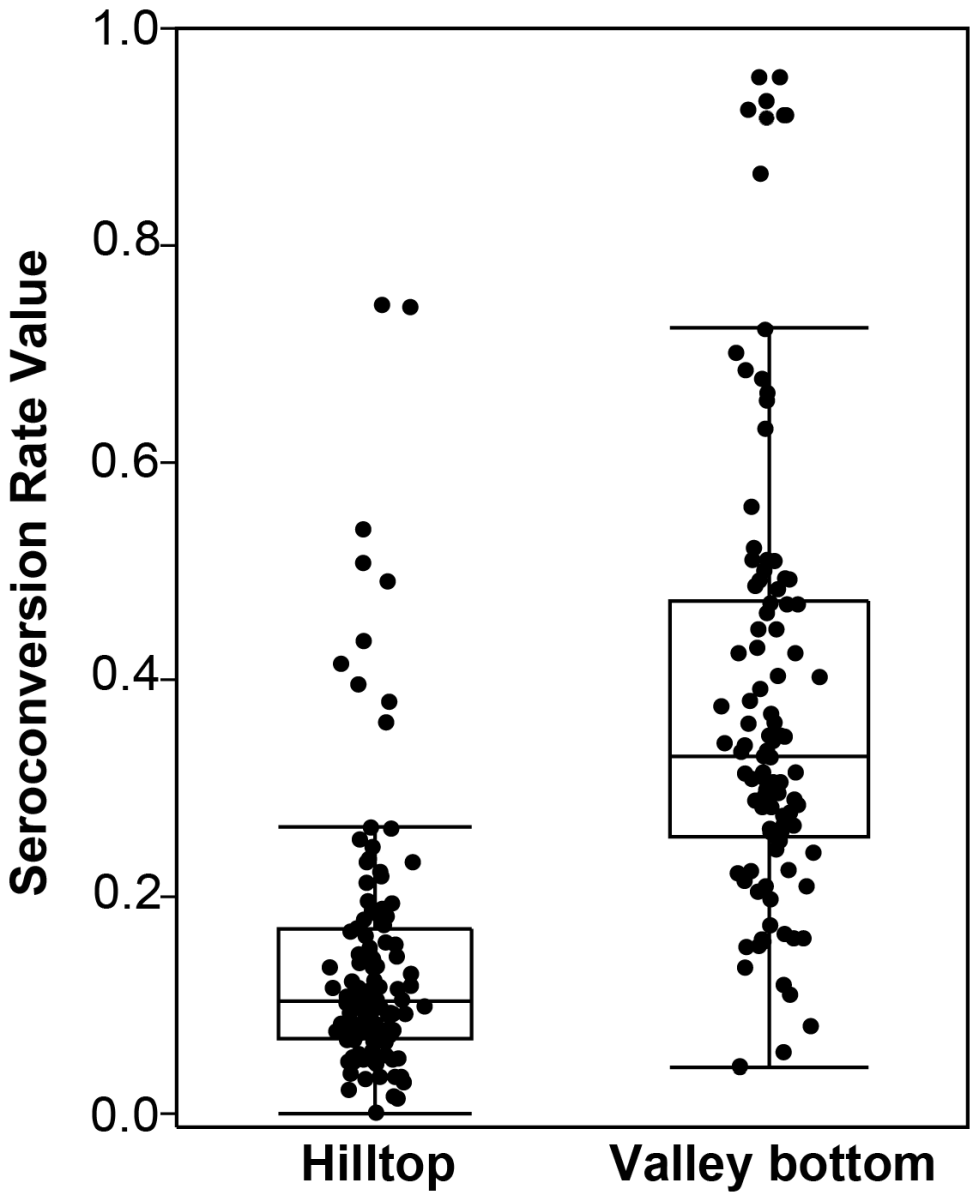
Whisker box plot of seroconversion rate (SCR) values of immunogenic polypeptides of *P. falciparum* for the hilltop and valley bottom sites. The SCR of 98 immunogenic polypeptides for which paired values were calculated (Y axis) is plotted against the site of serum sample collection (X axis). The lower and upper edges of the box indicate the first and the third quartiles, the line in the middle represents the median. The 1.5X interquartile range is indicated by the vertical line outside the boxes. Outlying values are indicated by dots outside boxes.

For the immunogenic polypeptides for which SCR and SRR values were calculated, a whisker box plot shows the comparison of SCR values between sites (Figure 6). In general, seroconversion rates were significantly lower in the hilltop (mean 0.12 [0.10 to 0.15]) than in the valley bottom (mean 0.38 [0.34 to 0.42]) (Mann-Whitney U test p<.0001). In this plot, outlier values represent immunogenic proteins that produce the highest SCR values in each site, and indicate antigens that require fewer exposures to the parasite to generate antibody responses even in the youngest age group. Table 2 identifies the immunogenic proteins with SCR values outside the 1.5X interquartile for each site. Amongst these proteins, three (the second exon of *var* gene PF3D7_0617400, the PF70 protein and Plasmodium exported protein (PHISTc) PF3D7_0801000) were common to both hilltop and valley bottom site lists, and had overlapping 95% confidence intervals, indicating that they produce similar dynamics of antibody response development in the populations of sites with differing parasite prevalences.

**Table 2:** *P. falciparum* polypeptides with highest (outlying) seroconversion rate values calculated from age-fitted seroprevalence curves for valley bottom and hilltop sites.

**Site**	**Gene ID**	**Gene product description**	**SCR**
**Valley bottom**	PF3D7_0617400	PfEMP1, *var* gene exon 2	0.96 (0.38-1.53)
	PF3D7_1002100	PF70 protein (PF70)	0.96 (0.38-1.53)
	PF3D7_0801000	Plasmodium exported protein (PHISTc), unknown function	0.93 (0.06-1.81)
	PF3D7_0108700	secreted ookinete protein, putative (PSOP24)	0.93 (0.41-1.44)
	PF3D7_1014100	conserved Plasmodium protein, unknown function	0.92 (0.33-1.51)
	PF3D7_1035700	duffy binding-like merozoite surface protein (DBLMSP)	0.92 (0.33-1.51)
	PF3D7_1036400	liver stage antigen 1 (LSA1), residues 185-434	0.87 (0.41-1.32)
**Hilltop**	PF3D7_0930300	merozoite surface protein 1 (MSP1), residues 1-870	0.75 (0.27-1.22)
	PF3D7_0617400	PfEMP1, *var* gene exon 2	0.74 (0.69-0.79)
	PF3D7_1239800	conserved Plasmodium protein, unknown function	0.54 (0.22-0.86)
	PF3D7_0801000	Plasmodium exported protein (PHISTc), unknown function	0.51 (0.11-0.91)
	PF3D7_1440500	allantoicase, putative	0.49 (-0.49-1.47)
	PF3D7_1002100	PF70 protein (PF70)	0.44 (0.14-0.73)
	PF3D7_1328300	conserved Plasmodium protein, unknown function	0.41 (0.16-0.67)
	PF3D7_1473000	conserved Plasmodium protein, unknown function	0.4 (0.04-0.75)
	PF3D7_0420700	erythrocyte membrane protein 1, PfEMP1 (VAR)	0.38 (0.22-0.54)
	PF3D7_0500800	erythrocyte membrane protein 2 (MESA)	0.36 (0.04-0.68)

Contrary to the proteins shown in Table 2 that generated similar SCR amongst the study sites, Table 3 presents proteins that produced significantly different SCR values based on their 95% confidence intervals. These antigens are less immunogenic than those presented in Table 2, and require a larger number of exposures to the parasite to generate antibody responses. In the valley bottom, where parasite prevalence is higher, the population becomes infected more often than the population from the hilltop, so conversion to seropositivity to these antigens stabilizes sooner than at the hilltop, generating higher SCR values. The differences with which antibody responses to these antigens develop between sites could be exploited in serological-surveillance for *P. falciparum* infections in low and moderate endemic areas. The parasite transmission level in a site could be estimated by measuring the SCR values for these antigens and comparing them amongst different sites, relating higher SCR values with higher parasite transmission levels and lower values with lower transmission levels.

**Table 3:** *P. falciparum* polypeptides that produced statistically different seroconversion rate values between valley bottom and hilltop sites.

**Gene ID**	**Gene Product Description**	**Hilltop**	**Valley bottom**
PF3D7_1121600	circumsporozoite-related antigen (CRA)	0.12 (0.05 - 0.19)	0.51 (0.39 - 0.63)
PF3D7_1035700	duffy binding-like merozoite surface protein (DBLMSP)	0.11 (0.05 - 0.17)	0.92 (0.33 - 1.51)
PF3D7_0108700	secreted ookinete protein, putative (PSOP24)	0.13 (0.01 - 0.25)	0.93 (0.41 - 1.44)
PF3D7_0206900.1	merozoite surface protein 5 (MSP5)	0.08 (0.05 - 0.11)	0.51 (0.26 - 0.76)
PF3D7_0501800	chromosome assembly factor 1 (CAF1), residues 692-1,239	0.09 (0.03 - 0.15)	0.47 (0.26 - 0.67)
PF3D7_0815500	conserved Plasmodium protein	0.05 (0.03 - 0.07)	0.34 (0.17 - 0.51)
PF3D7_1014100	conserved Plasmodium protein	0.14 (0.02 - 0.25)	0.92 (0.33 - 1.51)
PF3D7_0207000	merozoite surface protein 4 (MSP4), residues 1-164 (exon 1)	0.16 (0.01 - 0.30)	0.49 (0.38 - 0.60)
PF3D7_1002000	Plasmodium exported protein (hyp2)	0.07 (0.01 - 0.12)	0.33 (0.19 - 0.47)
PF3D7_0619000.1	conserved Plasmodium protein	0.05 (0.01 - 0.09)	0.37 (0.14 - 0.61)
PF3D7_1100800	Maurer's cleft two transmembrane protein (MC-2TM)	0.10 (0.04 - 0.16)	0.45 (0.21 - 0.68)
PF3D7_1134000	heat shock protein 70 (Hsp70-3)	0.13 (0.05 - 0.22)	0.40 (0.26 - 0.55)

Amongst the antigens with significantly different SCR values between sites are: the component of the parasitophorous vacuole membrane, circumsporozoite-related antigen (CRA), also known as exported protein-1 (Exp-1) [43,44]; the merozoite surface antigens Duffy binding-like merozoite surface protein DBLMSP [45] and the vaccine candidates merozoite surface protein-5 (MSP-5) [46] and MSP-4 [47], the product of conserved house-keeping genes chromosome assembly factor 1 CAF1 and heat shock protein 70-3 [48], a protein from the mosquito-associated stage ookinete, the putative secreted ookinete protein PSOP24, the erythrocyte-associated Maurer’s cleft 2-transmembrane protein [49], and several conserved Plasmodium proteins of unknown function.

## Discussion

In this work, we analyzed profiles of antibody binding to hundreds of *P. falciparum* (*Pf*) proteins produced by age-stratified sera from residents of two western Kenyan highland endemic areas with differing transmission intensities, during the dry and wet seasons. A major limitation of our study was the small sample size tested, especially in some of the sera cohorts collected in the hilltop locations. Nonetheless, many of our findings confirmed results of larger cohort studies, providing confidence that our results are representative of the wider picture. For example, we found that a large number of the proteins identified as immunogenic in our study had been previously reported as immunogenic in other epidemiological studies using protein microarray platforms [15,29,31,33] as well as other methods [50-56], to cite a few. 

We identified 107 proteins of *P. falciparum* that elicited antibody responses in naturally exposed populations of Kenyan highlands. When the antibody response to these proteins was compared between the higher parasite-prevalence site, valley bottom, and the low-prevalence site, hilltop, we observed no difference in the number of proteins recognized collectively by all ages. However, the intensity of antibody binding to the immunogenic proteins was associated with parasite prevalence level of the site of residence of donors, and was significantly lower in the hilltop than in the valley bottom. When breadth and intensity of response were analyzed in age-stratified manner, there were significant differences only between sera from those donors younger and those older than 5 years; children between the ages of 5 to 14 showed similar responses to adults above 15 years old. We also observed contrasting patterns in the temporal development of antibody response intensity amongst residents of the two sites: adults in the low transmission area showed the same intensity of antibody binding to *Pf* proteins as toddlers from the high transmission area.

With age-stratified serum samples we also calculated annual seroconversion rates (SCR) for a large number of the immunogenic proteins of *Pf* and compared those values between sites. We observed that the rate at which age-related seroprevalence to immunogenic proteins develops differs between sites, with SCR values generally being lower in the hilltop than in the valley bottom. However, based on confidence intervals, we identified a small number of proteins that produced similar SCR values between sites, as well as proteins that generated significantly different values between sites. 

It is widely recognized that the level of malaria immunity depends upon the parasite’s transmission intensity. In regions of stable transmission with high risk of infection, disease susceptibility is generally associated with age group below 5 years of age, while older children and adults are generally more protected from severe symptoms. However, in regions of unstable, medium or low transmission, all age groups are at risk of debilitating disease due to lack of protective immune responses [12,57]. In the western Kenya highlands, studies have shown that age, topography and drainage have a major impact on the levels of parasite prevalence and transmission [34,58,59]. The highlands have been considered a region of unstable transmission, but hot spots of high transmission are found, particularly in poorly drained river valley systems [34,60,61], where asymptomatic infections are more frequent and last longer than in higher altitudes [62], and represent an important parasite reservoir [63].

Earlier serological studies in this region of the Kenyan highlands have shown that the intensity of antibody responses to *P. falciparum* markers highly correlates with site-specific parasite prevalence, revealing differential exposure to infection between valley bottom and hilltop sites [20,60], and demonstrating that the risk of malaria transmission is highly heterogeneous in the area. The limited antigenic scope tested in those studies (MSP-1_19_ and CSP) was expanded to hundreds of proteins in the current work, but our findings echo those earlier conclusions. The intensity of antibody response to *P. falciparum* is significantly lower amongst residents of the low transmission hilltop site compared to the valley bottom where transmission is more stable. Although those previous studies did not compare the breadth of antibody responses between the sites, we found that there was significant difference in the number of antigens recognized by sera from the different sites but only amongst the youngest age group, confirming previous findings that repeated parasite exposure expands the repertoire of antibodies as children grow older [14,16], and is associated with protection from disease in adults [64].

The disparity in antibody responses between residents of areas of low or unstable and stable transmission, and its relationship with age, was also clearly demonstrated by our results showing that adults from the low transmission area had the same intensity of antibody responses as the youngest age group from the high transmission site. This finding is corroborated by another study comparing areas of unstable and stable transmission, where the youngest age group from a high transmission area and adults from the low transmission had similar antibodies levels to 4 antigens tested (CSP, LSA-1, AMA-1 and TRAP) [65]. 

Although the majority of our findings resonate with many other studies reporting the relationship between parasite prevalence and intensity of antibody response, the greatest advantage of our study was the ability to examine spatial and temporal variation of antibody responses from endemic populations against hundreds of *P. falciparum* proteins at once. The microarray platform provided us the opportunity to search for new targets of the immune responses and identify sero-epidemiological markers of infection that can be moved to a more widely deployable diagnostic platform and up-scaled in future studies.

Because serology reflects cumulative exposure to malaria, it can be a more dependable way to assess malaria endemicity than the short lifespan of the vector or the half-life of discrete infections, especially in low-transmission areas where mosquito distributions and parasite burden can vary widely. Seroconversion rates show strong correlation with entomological inoculation rates (EIR), the current gold standard for measuring malaria transmission intensity [17]. Age-fitted seroprevalence curves can be easily obtained from serological cross-sectional sampling of the population and the assay can be optimized for sensitivity to distinguish between areas of low and high transmission. For a serological-based approach to determine malaria transmission intensity, a combination of highly immunogenic antigens (to capture parasite exposure in low transmission areas) with less immunogenic antigens (to capture exposure in areas of high transmission, where seroprevalence to highly immunogenic antigens may reach 100% at early age), it is possible to assess the intensity of transmission in different settings [18,19,66,67]. However, identifying the best-suited markers for this application has been hampered by the limited number of antigens tested with this objective in mind.

We calculated seroconversion and reversion rates for almost 100 *P. falciparum* antigens using seroprevalence data from two areas of differing transmission intensities. We propose that by combining antigens that produce SCR values that are both high and similar between areas of different transmission levels (such as the second exon of Pf EMP1 PF3D7_0617400, Plasmodium exported protein PHISTc, or PF70), along with antigens that produced significantly different annual seroconversion rates between those sites (those shown in Table 3), it may be possible to design a sensitive immunoassay that allows for both the qualitative measure of exposure to *P. falciparum*, as well as the quantitative assessment of the relative level of malaria transmission intensity in an area. We propose that the antigens discussed here are potential candidates for sero-surveillance assays capable of distinguishing variations in transmission levels between areas, and estimating the relative parasite prevalence in each area separately, while still being sensitive enough to detect parasite exposure in areas of unstable and low transmission, such as the hilltop.

Our study benefited from state-of-the-art and relatively expensive technology, which is not commonly accessible to the malaria research community at large. However, an alternative or even complimentary method to identify candidates for new serological markers is to use bio-informatics data, such as from www.PlasmoDB.org, by searching gene ontology databases for specific characteristics. We showed that, amongst the 854 targets on the array, 107 were immunogenic and showed significant enrichment for particular categories in biological processes and cellular component gene ontology annotations, particularly those related to immune evasion, pathogenesis and expression of gene product on the host’s or parasite’s surface. When proteome microarray technology is not available, searching for genes in these categories may provide an additional method to choose gene products with serological survey potential.

The future direction for this work is to further evaluate the candidate antigens short-listed by SCR calculations, using larger sample cohorts and other high-throughput screening methods (such as ELISA or multiplexing Luminex assays) to validate their usefulness as serological markers of variation in *P. falciparum* transmission levels in endemic populations to distinguish sites of unstable and stable transmission. Additionally, we are extending this approach to identify serological markers useful worldwide, by working closely with investigators from the NIH-funded International Centers for Excellence in Malaria Research (ICEMR) in more than 20 countries and expanding our microarray platform to include 8,000 *P. falciparum* polypeptides. Similar studies need to be done in *P. vivax* endemic areas to identify antigens that can predict *P. vivax* exposure and reveal variations in malaria transmission intensity amongst sites. 

## Supporting Information

Figure S1 **Distribution of life stage of maximal expression of *P. falciparum* polypeptides represented on the P. falciparum Reactive Antigen microarray.** The percentage of the 854 polypeptides displayed on the microarray belonging to each of the parasite’s life stages is shown.
(TIF)

Figure S2 **Life stage and gene ontology distribution of *P. falciparum* proteins recognized as immunogenic by study sera.** **A**, percentage of immunogenic proteins with maximal expression in each life cycle stage of the parasite; **B**, percentage of immunogenic proteins belonging to each of the parasite’s cellular component; **C**, percentage of immunogenic proteins associated to each of the parasite’s biological processes.
(TIF)

Figure S3 **Plot of age-fitted seroprevalence to MSP-1 fragments by site of sample collection.** The percentage of individuals seropositive to the two MSP-1 fragments present of the microarray (Y axis) is plotted against the age of sample donors (X axis). Circles represent samples from the valley bottom (closed circles, seropositivity rate to MSP-1 residues 1-870; open circles, seropositivity rate to MSP-1 residues 752-1721); while triangles represent samples from the hilltop (closed triangles seropositivity rate to MSP-1 residues 1-870; open triangles, seropositivity rate to MSP-1 residues 752-1721).
(TIF)

Table S1 **List of *P. falciparum* proteins recognized as immunogenic by sera from naturally exposed individuals from western Kenyan highland sites on the protein microarray.**
(XLSX)

Table S2 **Enrichment analysis of annotated gene ontology Cellular Component, Biological Process and parasite life cycle stage of maximal expression for *P. falciparum* proteins recognized as immunogenic by sera from naturally exposed individuals from western Kenyan highland sites on the protein microarray.**
(XLSX)

Table S3 **Average and 95% confidence intervals for signal intensity of antibody binding to 107 immunogenic *P. falciparum* proteins by each study sera group.**
(XLSX)

Table S4 **Tukey-Kramer HSD clustering and pairwise comparison of z-scores of intensity of antibody binding to 107 immunogenic proteins for each of the sera groups.**
(XLSX)

Table S5 **Seroconversion (SCR) and Seroreversion (SRR) rate values for *P. falciparum* polypeptides considered immunogenic by the study sera, calculated for hilltop and valley bottom locations.**
(XLSX)
