# Supplementary figures and images for: Protein Microarray Analysis of Antibody Responses to Plasmodium falciparum in Western Kenyan Highland Sites with Differing Transmission Levels
Source: PLoS One. 2013 Dec 2;8(12):e82246. doi: 10.1371/journal.pone.0082246 (PMC3846730; doi:10.1371/journal.pone.0082246)

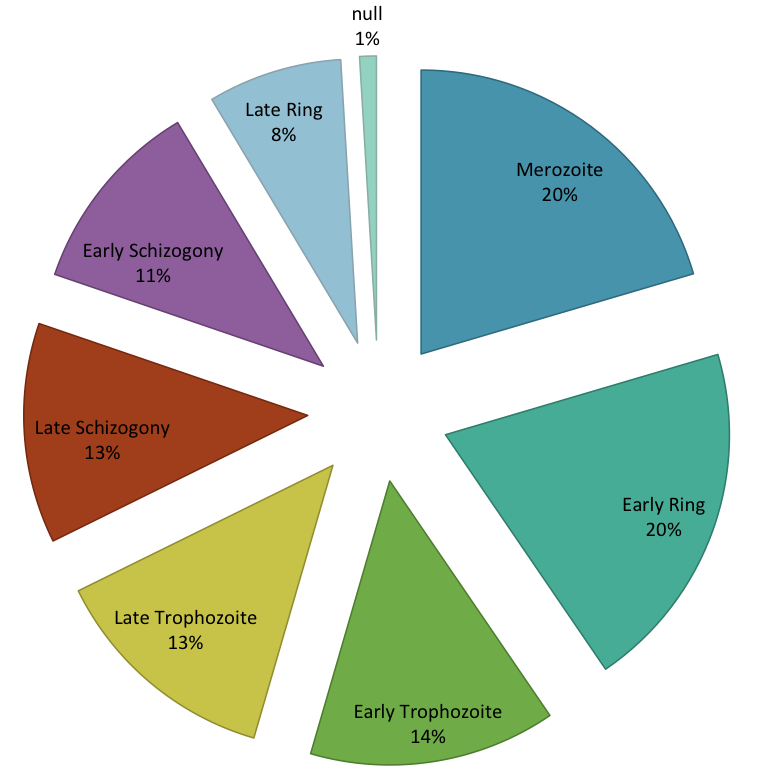

Supplement: Figure S1 — Distribution of life stage of maximal expression of P. falciparum polypeptides represented on the P. falciparum Reactive Antigen microarray. The percentage of the 854 polypeptides displayed on the microarray belonging to each of the parasite’s life stages is shown. (TIF) [file pone.0082246.s001.tif]

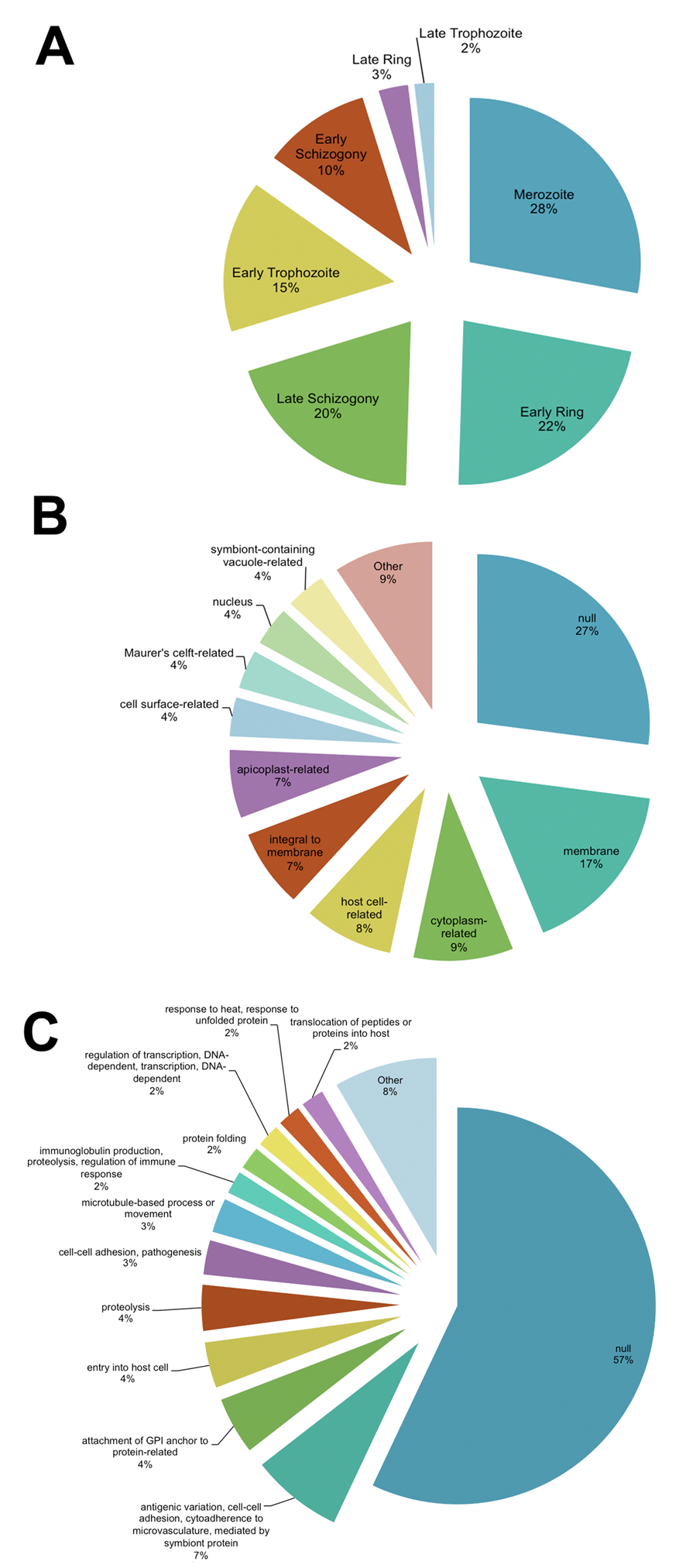

Supplement: Figure S2 — Life stage and gene ontology distribution of P. falciparum proteins recognized as immunogenic by study sera. A, percentage of immunogenic proteins with maximal expression in each life cycle stage of the parasite; B, percentage of immunogenic proteins belonging to each of the parasite’s cellular component; C, percentage of immunogenic proteins associated to each of the parasite’s biological processes. (TIF) [file pone.0082246.s002.tif]

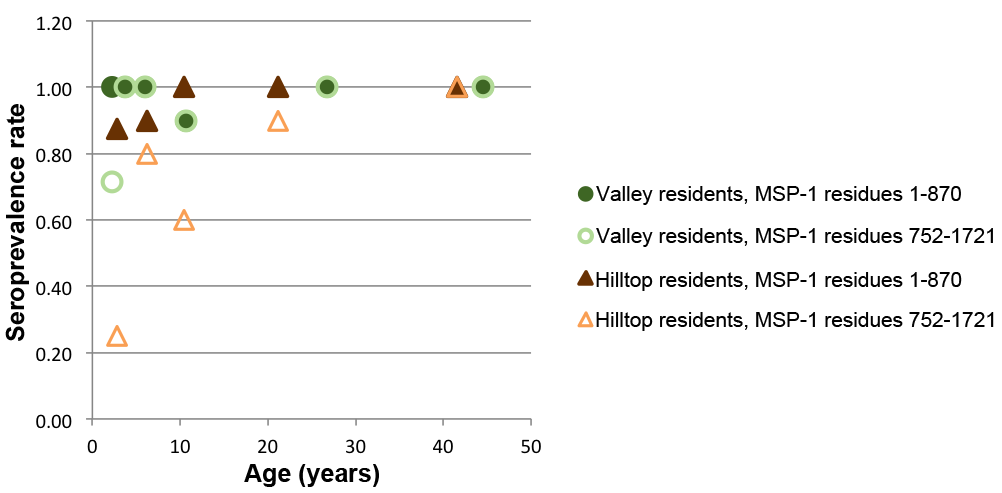

Supplement: Figure S3 — Plot of age-fitted seroprevalence to MSP-1 fragments by site of sample collection. The percentage of individuals seropositive to the two MSP-1 fragments present of the microarray (Y axis) is plotted against the age of sample donors (X axis). Circles represent samples from the valley bottom (closed circles, seropositivity rate to MSP-1 residues 1-870; open circles, seropositivity rate to MSP-1 residues 752-1721); while triangles represent samples from the hilltop (closed triangles seropositivity rate to MSP-1 residues 1-870; open triangles, seropositivity rate to MSP-1 residues 752-1721). (TIF) [file pone.0082246.s003.tif]
